# Progress in Diseases of the Blood

**Published:** 1901-02-09

**Authors:** 


### Progress in Diseases of the Blood.

**Blood Changes.**—Roger and Josué[1] find that the cells in the marrow undergo extraordinary multiplication when the individual is attacked by any infection. Thus, if pus cocci or their products are injected under the skin a proliferation of cells at the expense of the stored-up fat in the marrow takes place, and they are poured out into the blood. If this process is excessive it may occasionally go on to osteomyelitis, but in ordinary infections the marrow remains sterile. Antitoxins in like manner cause an increase in the production of red cells by the marrow. It is, however, curious to notice that McCrae found that an infection during leukæmia caused a diminution of the white cells in the blood though the myelocytes found here are supposed to come from the marrow. J. C. Bloodgood, of Johns Hopkins University, speaks of the diagnostic value of leucocytosis. Thus in appendicitis an increasing blood count shows danger, and if there are no physical signs but marked leucocytosis, there is probably a deep-seated abscess. In peritonitis some very high counts have been found, and if found in obstruction they indicate gangrene. In tubercular peritonitis there is no leucocytosis; so, too, in serous pleurisy, measles, uncomplicated typhoid or phthisis, mumps, malaria, and influenza. In all other septic and inflammatory diseases, according to T. L. Webb,[2] we find an increase. It need not be said how important this is in diagnosing obscure cases. However, it should be remembered that in very mild cases of *e.g.* pneumonia, or in very severe and fatal ones the reaction may be absent. In malignant disease and acute rheumatism we find there is a moderate leucocytosis, and the latter, like pneumonia, shows an excessive fibrin network in a film of blood as it dries. A severe hæmorrhage is shown by the reduction of the red cells to perhaps 3,000,000, or even 1,500,000, and of the hæmoglobin to 40 or 30 per cent. This may distinguish an internal hæmorrhage from peritonitis or cerebral concussion. In trichinosis we find a great excess of eosinophile cells. Again, the various types of severe anæmia can be distinguished by blood counts. Thus (*a*) in chlorosis the hæmoglobin is low—30 to 50 per cent.—while the red cells may be 3,000,000 or 4,000,000. In (*b*) pernicious anæmia this is reversed, the hæmoglobin is higher, but the red cells often sink to 1,000,000, and are much distorted in shape. Their average size is large and here alone very large nucleated red cells appear. (*c*) In secondary anæmias, *e.g.*, from cancer, the red cells are high, the hæmoglobin low, and leucocytosis is present. (*d*) In leukæmia the chief change is the enormous increase of white cells, especially of myelocytes, or lymphocytes with many nucleated red cells. (*e*) Finally, Hodgkins' disease in the early stages shows normal blood, though the spleen and glands are enlarged. Webb also praises Oliver's instrument for quickly reading off the number of red cells, and Hammerschlag's estimation of the hæmoglobin without a special instrument, provided dropsy is not present. In the latter method a drop of blood is let fall into a urinometer containing a mixture of chloroform and benzole. If it rises to the top more benzole is added, if it sinks, more chloroform. When stationary near the middle the specific gravity is taken and the following table gives the amount of hæmoglobin which corresponds to this specific gravity:—

| | | | |
|---|---|---|---|
| Sp. gravity. | 1,059 | = | 100 per cent. hœmoglobin. |
| | 1,056 | = | 80 |
| | 1,052 | = | 70 |
| | 1,049 | = | 60 |
| | 1,045 | = | 50 |
| | 1,041 | = | 40 |
| | 1,035 | = | 30 |
| | 1,030 | = | 20 |

Another labour-saving invention is Jenner's double stain, which not only differentiates the red and white cells and nuclei, but does away with "fixing" since the reagent is made up with absolute alcohol, which effectually fixes the films while staining them. For other details we must refer to Webb's paper, which is invaluable to the practical man. In a discussion at the Pathological Society,[3] stress was laid upon the low coagulability of the blood in urticaria and phthisis, and the presence of eosinophile cells in pemphigus and dermatitis herpetiformis, and Lorrain Smith explained chlorosis as having a great dilution or increase of the total volume of blood, while the red and white cells are actually increased, though the number in 1 c.c. is diminished. Carstairs Douglas[4] mentions a case of supposed chlorosis where a leucocytosis was found of 27,500 per c.m.m. This showed there must be some other cause, and the case finally proved to be a secondary anæmia, due to septic poisoning. In supposed early typhoid too the presence of a leucocytosis would cast doubt upon a positive Widal reaction. He has also never failed to find a leucocytosis in tubercular meningitis, the only tuberculous disease in which it is present; and the same test might be useful in deciding between pregnancy and an ovarian or fibroid tumour, for there is a large increase in the former case as well as after parturition, and two or three hours after meals containing proteids, and finally during infancy. Among the distinguishing points of splenic anæmia, on the other hand, is the normal or subnormal number of the white cells. J. G. Blackley[5] notices the absence of leucocytosis in measles and rothëln, which distinguishes them from scarlatina, syphilis, and diphtheria. He claims that it is present in influenza on the authority of Leichtenstern. In syphilis there is a change of the type of chlorosis, a decrease of the red cells, and a still greater one of the hæmoglobin, while in the secondary stage the cells show every variety of shape and size, and in the tertiary they approach the condition seen in pernicious anæmia. There is, too, in syphilis an increase of lymphocytes. In early stages Justus shows that the administration of mercury causes a fall of hæmoglobin in a day or so of 10 or 20 per cent., which is followed in two or three days more by a rise above the original amount. No other disease gives a similar reaction. M. C. G. Robertson and McKendrick[6] note the frequent absence of leucocytosis in small indolent malignant growths, while the change is marked in rapidly growing ones with metastases. An increase of the eosinophiles is found not only in asthma but also in sarcoma of bone, osteomalacia, psoriasis, pemphigus, exophthalmic goitre, and some diseases of the female genital organs.

The examination of fluid films is advocated by A. G. Phear,[7] who adds a little 40 per cent. alcohol coloured with methylene blue to a drop of blood on a slide, and then blows air through a pipette on the fluid so as to mix it thoroughly and examines it without any fixing. One advantage is that the delicate finely granular basophile cells, so often destroyed in films, are clearly shown in this way. They, like myelocytes, are numerous in splenic leukæmia, but rare in normal blood, and are one of the smallest cells known. A large form of lymphocyte with a feebly staining nucleus is frequent in acute leukæmia, where there is often a diminution of other red and white cells, with fever, stomatitis, and enlargement of the cervical and other glands. As to the origin of the cells of normal blood, he holds that the lymphocytes come from lymph tissue and develope into the large hyaline ones; the polynuclear ones probably from the marrow, and the origin of the eosinophiles and basophiles is unknown. A form of degeneration in red corpuscles has been described by Grawitz,[8] consisting in granulations staining with methylene blue. They are very numerous in pernicious anæmia and lead poisoning, and are also found in carcinoma and advanced leukæmia with septic processes. They seem to be absent in early phthisis, syphilis, Bright's disease, and hepatic cirrhosis. Osler and McCrae,[9] in discussing cancer of the stomach, think that megalo-blasts are rarely, if ever, found in the blood here; if in doubtful cases the red cells are below 1,000,000 the probability is in favour of pernicious anæmia, and variation of digestive leucocytosis, or of the form or number of the leucocytes is no guide in diagnosis.

**Pernicious Anæmia.**—R. C. Cabot[10] finds that the first symptoms are often muscular weakness, then a yellowish pallor, sometimes dyspnœa and soon after nausea. There may be vomiting without nausea, and intermittent gastric and intestinal disturbances. He examined 110 cases and all were fatal, the longest lasting five years. Treatment by arsenic, bone marrow, and other substances had no effect, but laxatives may be of some use. There are remarkable variations and intermissions in the disease. Spinal symptoms are common as well as pyrexia, albumen, hæmorrhages, enlargement of the liver and spleen, a great reduction of the red cells with little fall of hæmoglobin, the presence of megalocytes, oval and abnormally staining red cells, and a reduction of leucocytes especially of the polynuclear form. F. Henry[11] analyses five cases of the disease and refers to the difficulty of diagnosis from gastric cancer, for the general symptoms may be the same in both, but he thinks that the red cells in the former always fall before death to under one million, while in cancer they are relatively high. The high proportion of hæmoglobin in pernicious anæmia is due partly to the large size of the cells, and also to the number of microcytes, which are not counted though they contain much hæmoglobin; besides, at times large numbers of red cells are destroyed and their hæmoglobin is diffused in the serum, thus again raising the hæmoglobin compared with that in secondary anæmias. In conclusion he regards the condition as a reversion to the type of blood found in cold-blooded animals. This explains the number, size and shape of the red corpuscles and their hæmoglobin contents. It is remarkable, however, that, as Cabot states, the only secondary anæmia which resembles the pernicious form is that due to intestinal parasites, especially the bothriocephalus latus.

Another analysis of 20 cases is given by F. Billings,[12] who notices the constant tendency to gastro-intestinal disturbances among other symptoms. When improvement is taking place the hæmoglobin does not increase with the other elements and may at times be less than normal for the cells present. The nucleated red cells too diminish in such cases and may disappear at the height of the improvement. Most important of all are the megalo-blasts with irregular nuclei which are rarely, if ever, seen in secondary anæmias. Erben[13] notices the remarkable fall in the serum globulin of the blood, the other proteid bodies being little affected, while iron is present to a larger extent than the hæmoglobin will account for, and calcium and magnesium salts are increased.

[1] Brit. Med. Jour., Sept. 29. [2] Birm. Med. Rev., October. [3] Lancet, May 12. [4] Glasgow Med. Jour., July. [5] Monthly Homœop. Rev., December. [6] Glasgow Med. Jour., October. [7] Clin. Jour., Dec. 19, 26. [3] Med. Rec., Aug. 18. [9] New York Med. Jour. [10] Amer. Jour. of Med. Sci., August. [11] Amer. Jour. of Med. Sci., August. [12] Amer. Jour. of Med. Sci., November. [13] Amer. Jour. of Med. Sci., December.

